# Dynamic Gene Network Alterations and Identification of Key Genes in the Spleen During African Swine Fever Virus (ASFV) Infection

**DOI:** 10.3390/life15121844

**Published:** 2025-11-30

**Authors:** Jae-Beom Go, Vuong Nghia Bui, Duy Tung Dao, Ngoc Anh Bui, Jihye Cha, Hu Suk Lee, Dajeong Lim

**Affiliations:** 1 Department of Animal Resources Science, College of Agriculture and Life Sciences, Chungnam National University, Daejeon 34134, Republic of Korea; aea1241@cnu.ac.kr; 2 Virology Department, National Institute of Veterinary Research, Hanoi 100000, Vietnamddtung83@yahoo.com (D.T.D.); buingocanh_1980@yahoo.com (N.A.B.); 3 National Institute of Animal Science, Rural Development Administration, Wanju 55365, Republic of Korea; wischa91@korea.kr; 4 College of Veterinary Medicine, Chungnam National University, Daejeon 34134, Republic of Korea; hs.lee@cnu.ac.kr; 5 Department of Bio-AI Convergence, Chungnam National University, Daejeon 34134, Republic of Korea

**Keywords:** African swine fever virus (ASFV), spleen, gene co-expression network, RNA-seq, hub gene

## Abstract

ASFV is responsible for high mortality in domestic pigs and has caused substantial economic impact on the global swine industry due to herd losses, trade restrictions, and disease control measures. We analyzed publicly available spleen RNA-seq data from ASFV-infected pigs (*n* = 13 total samples), including 7 pre-infection (0 dpi), 4 samples at 2 days post-infection (2 dpi), and 2 samples at 5 dpi (5 dpi). Weighted gene co-expression network analysis (WGCNA) identified 19 modules; module–trait correlations revealed six modules associated with infection time. A co-expression module enriched for innate immune and antiviral response genes was strongly upregulated at 2 dpi, whereas a module enriched for ribosomal, translation, and metabolic process genes was broadly downregulated at 5 dpi. Protein–protein interaction analysis highlighted hub genes, including EPRS1 and USP7 within core cellular/translation programs and CMPK2 and ZBP1 within innate-immune signaling. Collectively, our results provide a network-level view of dynamic host responses and indicate coordinated shifts in immune and metabolic programs over time. These results identify CMPK2, ZBP1, EPRS1, and USP7 as hypothesis-generating hub gene candidates, warranting further validation to establish mechanistic roles and assess potential translational relevance.

## 1. Introduction

African swine fever (ASF) is a hemorrhagic disease of domestic and wild pigs with high transmissibility and near-inevitable mortality [1]. African swine fever virus (ASFV) is a large, enveloped double-stranded DNA virus classified in the family Asfarviridae, genus Asfivirus [2,3]. Since it was first reported in Kenya in 1921, ASF has become endemic in sub-Saharan Africa and, from the mid-20th century onward, has spread across Europe and Asia, repeatedly devastating pig populations and the pork industry [4,5]. Over the past decade, the emergence of ASF in new regions has underscored its ongoing threat. In 2018, ASFV reached China, where the virus decimated millions of pigs and disrupted global meat markets [6]. From China, ASFV rapidly crossed international borders; South Korea confirmed its first ASF outbreak in September 2019, leading to the culling of hundreds of thousands of animals to contain the spread [7,8,9]. Acute infection in naïve pigs presents high fever, profound lethargy, anorexia, and widespread hemorrhage and can rapidly progress to multi-organ failure, with case-fatality rates approaching 100% [10]. Even among survivors, subacute or chronic courses may result in prolonged viral shedding that complicates control [1]. Although two live-attenuated vaccines (NAVET-ASFVAC and AVAC ASF LIVE) have been conditionally approved for domestic use in Vietnam, no ASF vaccine has yet been widely licensed internationally, and no approved antiviral therapy is currently available for ASFV.

ASFV’s large (~170–190 kb) genome encodes more than 150 proteins that collectively subvert innate and adaptive immunity by interfering with pattern-recognition receptor pathways, suppressing type I interferon signaling, modulating apoptosis and autophagy, and reshaping cytokine responses [11,12,13,14,15].

Much of our mechanistic knowledge derives from cell-culture and ex vivo systems [16,17,18]. These studies have identified candidate receptors, defined the kinetics of early (pp220, p72) and late (pp62) viral proteins, and revealed viral antagonism of NF-κB and cGAS–STING signaling [19,20,21]. Transcriptomic profiling of infected primary macrophages similarly shows induction of CXCL10, TNF-α, IL-1β, and other innate mediators [22]. However, these approaches offer limited resolution on the temporal orchestration of host gene-expression programs throughout the course of infection in vivo.

The spleen is a key in vivo target organ during ASFV infection [23,24]. As a secondary lymphoid organ, it filters blood-borne pathogens, coordinates innate and adaptive immune activation, and serves as a principal replication site for ASFV [25]. Histopathology typically reveals hemorrhagic splenomegaly, extensive lymphocyte apoptosis, and loss of white-pulp architecture [25,26], reflecting the virus’s tropism for mononuclear phagocytes and highlighting the spleen’s value for interrogating systemic host responses. Nevertheless, most transcriptomic studies have focused on the early acute phase or on isolated splenic cell populations, leaving organ-level trajectories of splenic gene expression across infection poorly defined [25]. Consistent with this gap, prior reports show that the spleen exhibits the most severe pathology and the highest viral loads among swine tissues during ASFV infection, particularly at 3–7 dpi [24]. This observation highlights the relevance of time-resolved, whole-organ transcriptomic profiling. ASFV reaches the spleen rapidly following viremia, before disseminating to the liver, lungs, and lymph nodes [27]. These observations motivated our spleen-focused, time-resolved analysis.

RNA-seq enables high-resolution, whole-transcriptome profiling of ASFV-infected tissues [28]. Prior studies have cataloged hundreds to thousands of differentially expressed genes (DEGs) in the spleen, lymph nodes, and blood, highlighting Toll-like receptor signaling, apoptosis, and cell-cycle regulation [29,30]. However, most analyses examined a single time point or simple infected-versus-control contrasts, and ex vivo macrophage studies typically captured only the first 24–48 h. Consequently, the coordinated, time-dependent waves of antiviral defense, inflammatory cascades, and tissue-repair programs in vivo remain incompletely defined. Given that ASFV replication and host responses unfold over days—ranging from incubation at 4–19 days to systemic spread by 5–7 dpi [31]—time-resolved transcriptomics is crucial to uncovering induction–repression waves and key regulatory circuits that static DEG lists miss [32].

Network-based approaches such as weighted gene co-expression network analysis (WGCNA) group genes into co-regulated modules, revealing higher-order organization and pinpointing hub genes implicated in disease progression [33]. Integrating co-expression modules with protein–protein interaction (PPI) networks further highlight genes central to molecular crosstalk [34].

While single-cell studies [24] provide high-resolution insights into cell-type dynamics, our study emphasizes time-resolved, bulk tissue gene-expression networks in the spleen, offering a complementary perspective. To this end, we analyzed RNA-seq data from porcine spleen collected before inoculation (day 0; uninfected controls) and at 2 and 5 days post-infection (dpi). Notably, acute ASFV infection typically leads to death within 7 dpi [35]. Consequently, our inclusion of the 5 dpi time point provides critical insight into the later stages of infection, just before mortality. This time-series analysis offers comprehensive insights into the entire progression of ASFV infection, from the early phase to the late, fatal stage. We constructed gene co-expression networks to define time-dependent expression patterns, performed KEGG and Gene Ontology (GO) enrichment to characterize module functions, mapped module genes onto STRING database (v12.0, https://string-db.org, accessed on 15 August 2025)/Cytoscape plug-in (v0.1) and CluePedia (v1.5.10) to identify hub genes, and correlated hub-gene expression with immune markers. This integrative, network-based framework moves beyond static DEG catalogs and delineates how antiviral, inflammatory, apoptotic, and metabolic programs are coordinated during ASFV infection, highlighting network-central genes and providing a systems-level vantage that complements cell-type-specific findings. This integrative, network-based framework resolves infection-stage–specific co-expression networks and regulatory hubs, clarifying how early innate activation at 2 dpi transitions to broader inflammatory and tissue-remodeling programs by 5 dpi and providing a mechanistic context for splenic pathology progression.

## 2. Materials and Methods

### 2.1. Data Source and Original Experimental Design

This study did not involve new animal experiments. All RNA-seq data analyzed here were obtained from the publicly available NCBI SRA repository (BioProject PRJNA230240; data generated in Miyazaki, Japan), originating from the ASFV infection study cited as reference [27].

In that study, pigs were screened for major swine pathogens and experimentally infected with the ASFV strain VNUA/HY/Vietnam (GenBank accession MK554698) under approved institutional animal care and biosafety protocols. Spleen tissues were collected at pre-infection baseline (0 dpi, *n* = 7), at 2 days post-infection (2 dpi, *n* = 4), and at 5 days post-infection (5 dpi, *n* = 2), totaling 13 samples. The present study re-analyzes these publicly available spleen RNA-seq data to characterize temporal transcriptional responses and gene regulatory network dynamics following ASFV infection.

### 2.2. RNA-Seq Data Processing

Raw FASTQ files were downloaded from NCBI SRA (BioProject: PRJNA230240). Quality control was performed using Trimmomatic v0.39 [36] with adapter and quality trimming (seed mismatches = 2, palindrome clip = 30, simple clip = 10; LEADING:3, TRAILING:3, SLIDINGWINDOW:4:15; MINLEN:36). Clean reads were aligned to the Sus scrofa 11.1 reference genome with HISAT2 v2.2.1 [37]. Gene-level counts were generated using featureCounts v2.0.1 (Subread package; https://subread.sourceforge.net/; accessed on 15 August 2025) with Ensembl Release 113 annotations (Sus scrofa 11.1).

### 2.3. Weighted Gene Co-Expression Network Construction in Spleen Tissue During ASFV Infection Time

**Figure 1:**
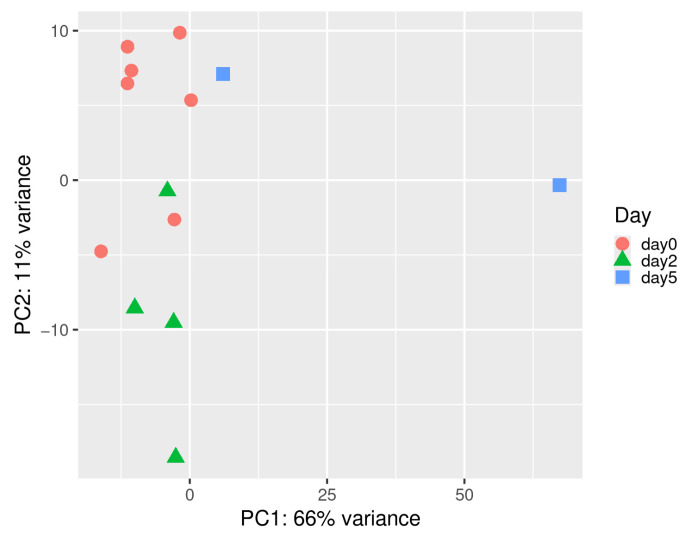
Principal Component Analysis (PCA) of gene expression profiles in spleen tissue across ASFV infection time points. PCA was performed using vst-normalized gene expression data from spleen samples collected at 0, 2, and 5 dpi. Each point represents an individual sample, with color and shape indicating the time point (day 0: red circles, day 2: green triangles, day 5: blue squares). The first two principal components (PC1 and PC2) explain 66% and 11% of the total variance, respectively.

**Figure 2:**
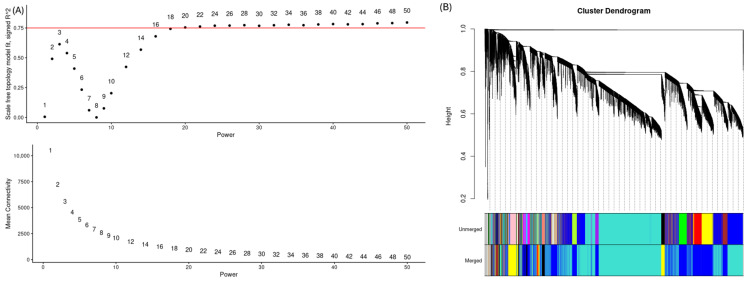
Network construction and module detection in WGCNA. (**A**) Analysis of network topology for various soft-thresholding powers. The top panel shows the scale-free topology fit index (R^2^) as a function of the power, with a horizontal red line indicating R^2^ = 0.75. The soft-thresholding power β = 16 was selected as the lowest power achieving R^2^ ≥ 0.75. The bottom panel displays the corresponding mean connectivity at each power. (**B**) Gene clustering dendrogram based on hierarchical clustering of the topological overlap matrix. Modules were identified using dynamic tree cutting (Unmerged) and merged based on eigengene similarity (Merged). The colored rows below the dendrogram correspond to the module assignments.

We analyzed 13 samples (pre-infection baseline, day 0; uninfected controls: *n* = 7; 2 dpi: *n* = 4; 5 dpi: *n* = 2). Genes with raw counts ≥15 in ≥5 samples were retained. Counts were variance-stabilized using DESeq2 (vst). PCA confirmed clustering by time point (Figure 1). We built co-expression networks with WGCNA, selecting the soft-threshold power using pickSoftThreshold; β = 16 was the lowest value yielding a scale-free topology fit index >0.85 (Figure 2A). Modules were identified by hierarchical clustering with blockwiseModules. The topological overlap matrix (TOM) produced dendrograms before/after merging (Figure 2B). Modules with eigengene correlation ≥0.75 were merged using a height cutoff of 0.25. Hub genes were identified with chooseTopHubInEachModule based on intramodular connectivity.

### 2.4. Functional Enrichment Analysis of Gene Co-Expression Modules

We identified 19 modules (excluding grey). For each non-grey module, KEGG and Gene Ontology (GO: BP/MF/CC) enrichment were performed using clusterProfiler with Benjamini–Hochberg (BH) multiple-testing correction; FDR < 0.05 was considered significant. The background gene set was all genes passing the expression filter.

### 2.5. Pathway and Protein–Protein Interaction (PPI) Network Analysis

Modules significantly associated with infection time were further analyzed. Enriched KEGG pathway networks were visualized using ClueGO v2.5.10 and CluePedia in Cytoscape v3.10.3 (right-tailed hypergeometric test; BH correction; *p* < 0.05; kappa ≥ 0.4). Module genes were mapped to STRING v12.0 PPI networks. Node prioritization used cytoHubba (Maximal Clique Centrality, MCC); the top 20 nodes per module were designated as key hubs [38].

### 2.6. Statistical Analysis

All analyses were performed in R (v4.2.2) using the following packages: DESeq2, WGCNA, clusterProfiler, org.Ss.eg.db, tidyverse, ggplot2/ggrepel/ggpubr, gridExtra, and enrichplot. Network visualization and prioritization were conducted in Cytoscape (v3.10.3) with cytoHubba, ClueGO, and CluePedia. All computations were performed at the individual-sample level; no RNA pooling was performed. Low-quality samples and genes were screened using goodSamplesGenes, and genes with raw counts ≥15 in ≥5 samples were retained. Variance-stabilizing transformation (VST) from DESeq2 was applied prior to downstream analyses, and VST values were used for PCA and WGCNA to stabilize mean–variance dependence. Module–trait associations were assessed by Pearson correlation between module eigengenes and time point indicators (0/2/5 dpi). *p*-values were calculated using corPvalueStudent (*n* = 13) and adjusted across the module × trait matrix using the Benjamini–Hochberg procedure, with FDR < 0.05 considered significant. Functional enrichment for KEGG and Gene Ontology (BP/MF/CC) was conducted using clusterProfiler, employing right-tailed hypergeometric tests and BH correction (background set: all genes passing the expression filter), again applying FDR < 0.05 as the significance threshold. Hub genes were summarized in two complementary ways: (i) expression-based hub genes were defined by module membership (kME ≥ 0.9), calculated as the correlation between each gene and the module eigengene; and (ii) network-based hub genes were defined using Maximal Clique Centrality (MCC) in STRING-derived protein–protein interaction networks; for the latter, the top 20 MCC-ranked genes per module were reported.

## 3. Results

### 3.1. Sequencing Quality and Read Statistics

As shown in Table 1, an average of 36,484,500 raw read pairs (standard deviation: 3,403,084) were generated per sample. After quality control using Trimmomatic, an average of 35,227,179 read pairs remained, indicating high overall sequencing quality with minimal read loss. On average, only 33,691 reads (approximately 0.09%) were discarded per sample, suggesting that the majority of the sequences passed the filtering criteria.

### 3.2. Construction of Co-Expression Network in Spleen Tissue and Correlation Analysis with Infection Time Points

**Figure 3:**
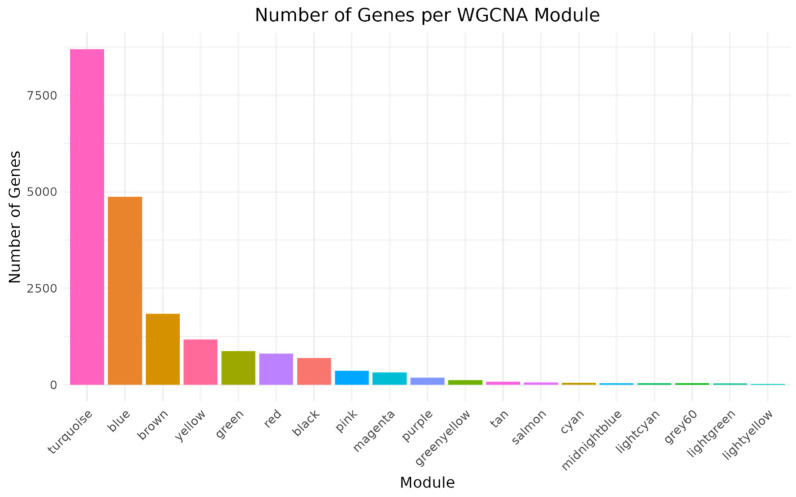
Gene Count per Co-expression Module. Bar plot showing the number of genes assigned to each co-expression module identified by WGCNA. Modules are ordered by size along the x-axis, and the y-axis indicates the gene count per module.

**Figure 4:**
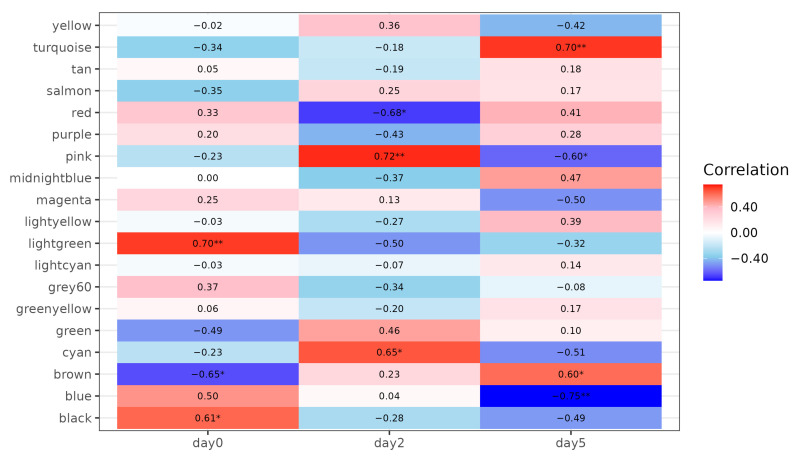
Correlation matrix heatmap illustrates the relationships between WGCNA-derived gene co-expression modules and ASFV infection stages (0, 2, 5 dpi). Colors indicate the strength and direction of correlation between each module and time point, with red representing positive and blue negative correlations. Asterisks within cells denote significance levels: * *p* < 0.05, ** *p* < 0.01.

Of the 34,200 genes, those with read counts less than 5 in three or more of the 13 samples were filtered, resulting in 21,024 genes for gene co-expression network analysis. Principal component analysis (PCA) based on these genes revealed that samples were distinctly clustered by infection time point (0, 2, 5 dpi), with day 5 samples separating along the PC1 axis (Figure 1). PC1 and PC2 explained 66% and 11.1% of the total variance, respectively, indicating a strong temporal effect of ASFV infection on gene expression. We used WGCNA to identify 19 co-expressed gene modules exhibiting distinct expression patterns over time (Figure 2 and Figure 3). We then performed module-trait correlation analysis using Pearson correlation to analyze the relationship between each module and the infection time point (Figure 4). Among the 19 modules, six gene co-expression modules (blue, pink, cyan, turquoise, red, brown) showed statistically significant correlations (*p* < 0.05) with at least one infection time point, suggesting a potential association with gene expression changes following ASFV infection. The modules showed significant changes across time points, suggesting dynamic transcriptional reprogramming in response to ASFV infection. Module-trait correlation analysis revealed that the pink (r = 0.72, *p* = 0.0057) and cyan (r = 0.65, *p* = 0.01) modules were positively correlated with early ASFV infection (2 dpi), indicating upregulation during this stage of infection. In contrast, the red module showed a significant negative correlation with early infection (r = −0.67, *p* = 0.01). At the late stage of infection (5 days), the brown (r = 0.60, *p* = 0.03) and cyan (r = 0.70, *p* = 0.007) modules showed positive correlations, whereas the blue (r = −0.75, *p* = 0.003) and pink (r = −0.60, *p* = 0.031) modules showed negative correlations. Among these 19 modules, six (pink, cyan, red, turquoise, brown, and blue) showed significant correlations with at least one infection time point and were selected for functional interpretation. Functional enrichment of these six infection-associated modules is summarized in Table 2. In the text, we highlight only the major biological themes (early antiviral/innate at 2 dpi; regulatory/TNF-related downregulation; late adhesion/translation remodeling at 5 dpi), while detailed KEGG/GO terms and representative genes are reported in Table 2.

### 3.3. Activation of Innate Antiviral Response–Associated Co-Expression Modules at 2 dpi

At this time point, two gene co-expression modules (cyan and pink) were significantly activated (cyan: r = 0.65, *p* = 0.01; pink: r = 0.72, *p* = 0.0057). In the cyan module, KEGG analysis indicated enrichment in pathways related to Huntington’s disease, selenocompound metabolism and amyotrophic lateral sclerosis (ALS) (Table 2).

**Figure 5:**
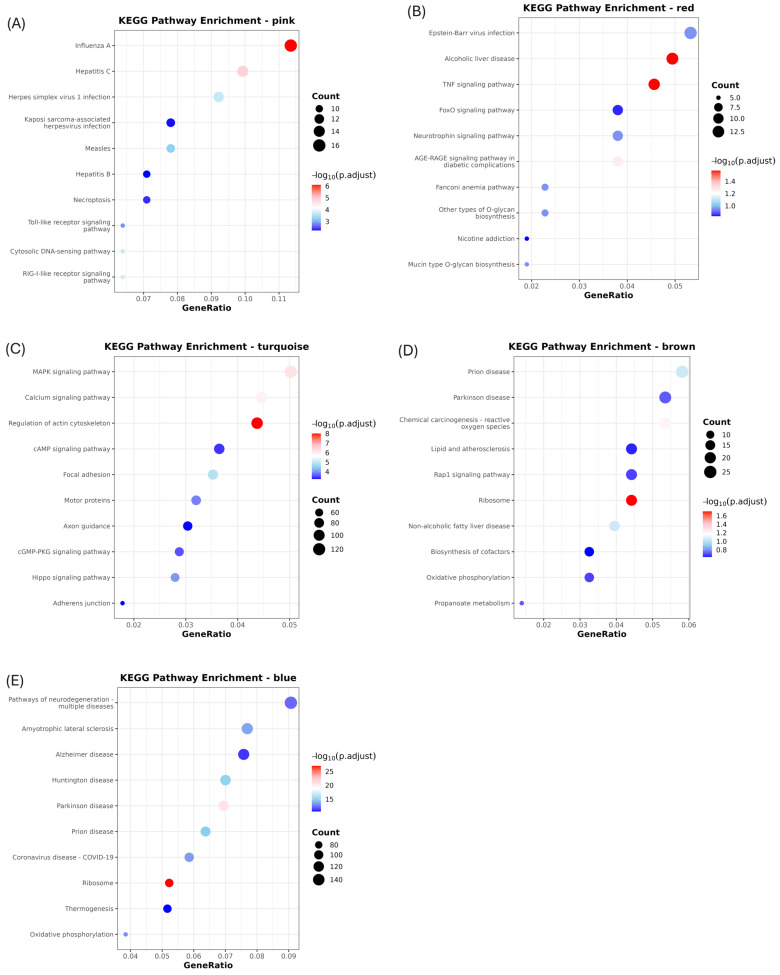
KEGG pathway enrichment analysis of genes from co-expression modules identified by WGNCA. (**A**) KEGG pathway enrichment of genes in the pink module. (**B**) KEGG pathway enrichment of genes in the red module. (**C**) KEGG pathway enrichment of genes in the turquoise module. (**D**) KEGG pathway enrichment of genes in the brown module. (**E**) KEGG pathway enrichment of genes in the blue module. Each dot represents a significantly enriched KEGG pathway (adjusted *p*-value < 0.05). The x-axis indicates the gene ratio (the proportion of genes in the module involved in a given pathway), while the size of each dot reflects the number of genes associated with that pathway. The color gradient represents the adjusted *p*-value, with darker red indicating higher significance. Pathways were identified using enrichment analysis of genes within each module, and only the top significant terms are shown.

**Figure 6:**
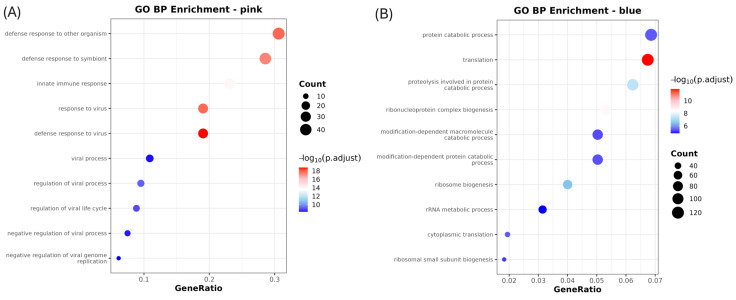
Gene Ontology (GO) Biological Process (BP) enrichment analysis of blue and pink modules. (**A**) GO BP enrichment terms for the pink module. (**B**) GO BP enrichment terms for the blue module. Each dot represents a significantly enriched GO term (adjusted *p*-value < 0.05). The x-axis indicates the GeneRatio (number of genes associated with the term divided by total input genes), while the dot size reflects the number of genes enriched for each term (Count). Color intensity represents the adjusted *p*-value (p.adjust), with red indicating stronger statistical significance.

**Figure 7:**
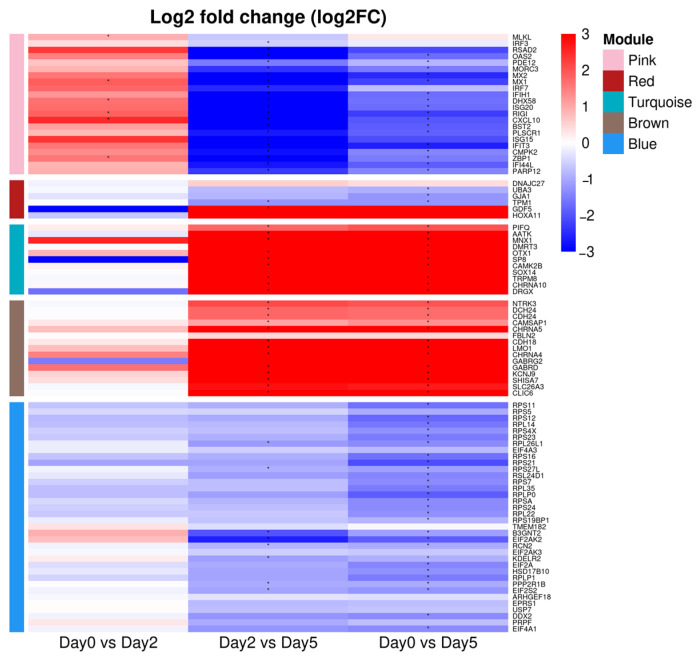
Log2 fold change(log_2_FC). Time-course expression heatmap of module genes. Rows are genes grouped by module in the order Pink, Red, Turquoise, Brown, Blue (left color bar), and columns show the three contrasts Day0 vs. Day2, Filling in the blanks is correct. Thank you for the good point.Day2 vs. Day5, and Day0 vs. Day5. Colors encode log_2_ fold change (red = up-regulation, blue = down-regulation); values are clipped to ±3 for comparability across genes. Asterisks mark cells with FDR-adjusted *p* (padj) < 0.05. A positive log_2_FC indicates higher expression at the latter time point of the contrast (e.g., Day2 vs. Day0 > 0 means higher at Day2).

In pink module, KEGG analysis showed significant enrichment in immune-related pathways such as Influenza A, Hepatitis C, necroptosis, RIG-I-like receptor signaling, Toll-like receptor signaling, and the cytosolic DNA sensing pathway (Figure 5A, Table 2). GO terms such as defense response to virus, innate immune response, and response to were also significantly enriched (Figure 6A, Table 2). Of the three GO terms above, the common genes identified were *MLKL*, *IRF3*, *RSAD2*, *OAS2*, *OASL*, *PDE12*, *MORC3*, *MX2*, *MX1*, *IRF7*, *IFIH1*, *TRIM65*, *DHX58*, *ISG20*, *RIGI*, *CXCL10*, *BST2*, *PLSCR1*, *ISG15*, *and IFIT3* (Table 2; Figure 7).

**Figure 8:**
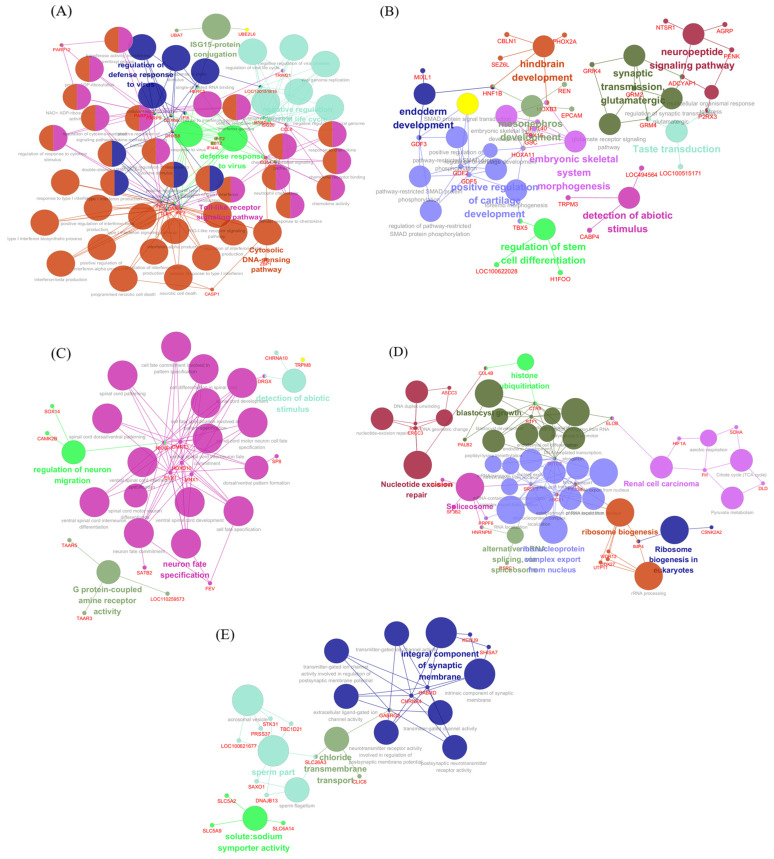
Functional enrichment networks of genes in the pink and blue modules using Cluepedia. (**A**) Functional enrichment network of the pink module. (**B**) Functional enrichment network of the red module. (**C**) Functional enrichment network of the turquoise module. (**D**) Functional enrichment network of the brown module. (**E**) Functional enrichment network of the blue module. Nodes represent functionally enriched terms, and the node size is proportional to the number of genes in the corresponding term. Nodes with the same color represent coherent functional clusters. Edges (lines) connect terms that share common genes.

To further dissect the functional architecture of the pink module, we conducted an integrated pathway and interaction analysis using ClueGO and CluePedia (Figure 8A). Genes with node degrees ranging from 10 to 35 based on Maximal Clique Centrality (MCC) for the construction of a biological functional network. CluePedia-based network analysis revealed tightly connected clusters associated with ISGylation, double-stranded RNA recognition, viral replication suppression, and ADP-ribosylation. Genes such as ZBP1, IFI44L, and PARP12 were located at the intersection of multiple functional clusters.

**Figure 9:**
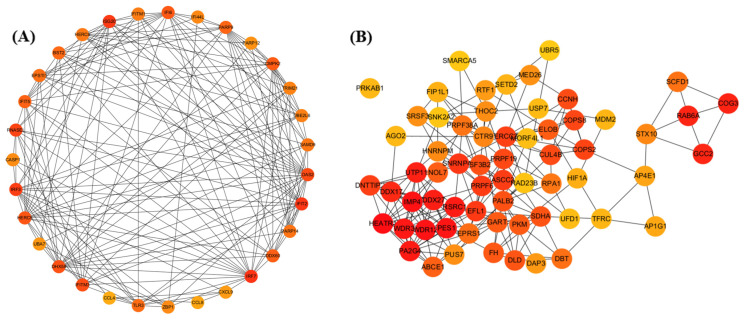
Protein–protein interaction (PPI) networks of the pink and blue modules. (**A**) PPI network of genes in the pink module. (**B**) PPI network of genes in the blue module. Nodes represent genes, and edges indicate predicted or known protein–protein interactions obtained from the STRING database. Node color represents network centrality, with warmer colors (orange to red) indicating higher connectivity (e.g., higher degree or MCC score).

To identify core regulatory genes within the pink module, we performed protein–protein interaction (PPI) analysis using all pink module genes in the STRING database. A total of 191 genes were used to construct the network, and genes with node degrees between 10 and 35 were selected to enhance biological specificity (Figure 9A). Among them, CMPK2 was identified as the top hub gene, being the only gene with a module membership(kME) value exceeding 0.9.

### 3.4. Downregulation of Gene Co-Expression Modules at 2 dpi

The red module at 2 dpi showed a strong negative eigengene correlation and was enriched for immune-regulatory and inflammatory pathways, including. In the red module, KEGG pathway analysis indicates TNF signaling and related processes (Figure 5B, Table 2). Several transcripts annotated to TNF-associated signaling (e.g., DNAJC27, UBA3, GJA1, TPM1) exhibited reduced expression at this time point (Figure 7). To further resolve the functional organization of this module, we performed network-based enrichment using ClueGO/CluePedia on the top 500 genes ranked by module membership (Figure 8B). This analysis identified two principal functional clusters. The first corresponded to a TGF-β/SMAD-associated developmental program, with GDF5 and HOXA11 occupying central node positions. The second cluster comprised neurodevelopmental genes associated with hindbrain patterning, indicating coordinated modulation of developmental regulatory pathways at 2 dpi. These results indicate that, at 2 dpi, alongside strong antiviral activation in other modules, a subset of immune–developmental signaling pathways undergo coordinated downregulation. This suggests that the early host response involves not only immune activation, but also regulatory mechanisms that may modulate inflammatory tone and tissue homeostasis prior to the onset of overt pathological damage.

### 3.5. Modules Activated During Later Infection

During this stage, the turquoise and brown modules showed significant positive correlations with infection status (turquoise: r = 0.70, *p* = 0.007; brown: r = 0.60, *p* = 0.03).

In the turquoise module, Kegg pathway indicates that MAPK signaling, calcium signaling, cAMP, cGMP–PKG signaling, and Hippo signaling (Figure 5C, Table 2). Additionally, pathways related to cellular structure and motility, such as focal adhesion, adherent, motor protein activity, and axon guidance were also enriched (Table 2).

To explore the biological profile of the turquoise module, we ran ClueGO and CluePedia on the top 500 high-membership genes (Figure 8C). The CluePedia network resolved a compact core for neuron fate and spinal cord patterning, with MNX1, DMRT3, OTX1, and SP8 at the center. First, a peripheral cluster related to the regulation of neuron migration was observed, represented by CAMK2B and SOX14. Second, a cluster associated with the detection of abiotic stimuli included TRPM8, CHRNA10, and DRGX. Third, a cluster related to amine-responsive GPCR signaling was identified.

In the brown module, the KEGG pathway indicates only ribosome enrichment (Figure 5D). The genes included in this pathway were NTRK3, DCH24, CDH24, CDH13, CAMSAP1, CHRNA5, FBLN2, CDH18, and LMO1, and their expression levels were found to increase on 5 dpi.

We also analyzed the top 500 high-membership genes using ClueGO and CluePedia (Figure 8D). A cluster containing CHRNA4, GABRG2, GABRD, KCNJ9, and SHISA7 was enriched in ligand-gated ion channel terms. A second cluster, represented by SLC26A3 and CLIC6, indicated chloride transport.

### 3.6. Modules Showing Decreased or Changing Expression During Infection

At 5 dpi, a general downregulation of gene expression was observed in both the blue and pink modules (pink: r = −0.60, *p* = 0.031; blue: r = −0.75, *p* = 0.003). Notably, the blue module showed a gradual decline in expression over time, while the pink module was transiently activated during the early phase of infection but subsequently suppressed at the later stage.

In blue module, KEGG and GO analyses revealed significant enrichment in oxidative phosphorylation, ribosome function, thermogenesis, and pathways associated with neurodegenerative diseases such as Alzheimer’s, Parkinson’s, and Huntington’s disease (Figure 5E, Table 2). GO Biological Process analysis further highlighted key cellular functions such as protein translation, ribosome biogenesis, rRNA metabolic process, and ribonucleoprotein complex biogenesis (Figure 6B, Table 2).

Among the genes involved in protein synthesis and translation, those that showed decreased expression at 5 dpi were RPS11, RPS5, RPS12, RPL14, RPS4X, RPS23, RPL26L1, EIF4A3, RPS16, RPS21, RPS27L, RSL24D1, RPS7, RPL35, RPLP0, RPSA, RPS24, RPL22, and RPS19BP1. In addition, genes related to mitochondrial energy production that were downregulated at 5 dpi included TMEM182, B3GNT2, EIF2AK2, RCN2, EIF2AK3, KDELR2, EIF2A, HSD17B10, RPLP1, PPP2R1B, EIF2S2, and ARHGEF18.

CluePedia analysis of the blue module hub genes (MM > 0.9, 10 < MCC < 35) highlighted various epigenetic and post-transcriptional regulatory functions, including ribosome biogenesis, RNA transport, splicing, histone modification, and mitochondrial energy metabolism (Figure 8E). Key genes, including USP7, WDR12, and HNRNPM, formed the network’s core. Notably, USP7 was significantly reduced at 5 dpi in the blue module (Figure 7). STRING-based PPI analysis identified RNA splicing and translation regulators—including EPRS1, USP7, DDX2, PRPF, and EIF4A1 (Figure 9B).

The pink module was activated at 2 dpi but became significantly suppressed by 5 dpi (r = −0.60, *p* = 0.031). Key hub genes within this module, such as CMPK2 and ZBP1, were significantly downregulated at 5 dpi (Figure 9).

## 4. Discussion

### 4.1. Early Macrophage-Driven Antiviral and Inflammatory Activation at 2 dpi

At 2 dpi, during the early stage of ASFV infection, the host rapidly initiated both innate immune responses and metabolic stress reactions. At 2 dpi, cyan and pink modules showed biological responses to viral infection. Cyan modules showed significant functional enrichment in pathways related to oxidative stress and mitochondrial dysfunction.

Huntington’s disease and amyotrophic lateral sclerosis (ALS) are neurodegenerative disorders known to be closely associated with mitochondrial reactive oxygen species (mtROS) production and cellular metabolic disturbances [39]. These results suggest that ASFV infection may induce intracellular oxidative damage and metabolic imbalance early in the disease, contributing to tissue damage and the progression of pathogenesis [40].

These genes are deeply involved in antiviral and innate immunity, and their expression increased at day 2 (Figure 7). These results suggest that the host rapidly recognizes the virus and activates innate immune signaling pathways early in infection.

Previous studies showed cGAS–TLR9 sensing triggers STING/TBK1/IRF3 and NF-κB, ultimately inducing ISGs via JAK–STAT [20,39]. KEGG and GO analyses enriched the innate immune pathway, and CMPK2, one of the interferon-stimulated genes in the pink module, was upregulated on day 2 (Figure 7) [41]. These observations support early activation of innate immunity in the co-expression network.

ISGylation is known as an antiviral mechanism in various viral models, and some viruses have evolved strategies to evade replication suppression by counteracting host ADP-ribosylation mechanisms [42].

Genes such as ZBP1, IFI44L, and PARP12 were located at the intersection of multiple functional clusters and are known to play important roles in pathogen recognition and immune signaling (Figure 7). ZBP1 is a key cell death gene that detects intracellular double-stranded RNA or Z-form nucleic acids during viral infection and induces necroptosis and apoptosis [43]. PARP12 is a representative ISG that inhibits viral replication via ADP-ribosylation, while IFI44L is an interferon-inducible gene that is upregulated upon viral infection [44,45].

CMPK2 (KME = 0.943) is a mitochondria-localized ISG that couples TLR/IFN signals to mtDNA synthesis and NLRP3 inflammasome activation in macrophages, aligning with inflammatory injury mechanisms in infection [46]. Beyond inflammation, CMPK2 serves as a broad antiviral restriction factor, suppressing Zika viral translation and curbing multiple coronaviruses [47,48]. Although ASFV encodes antagonists of cGAS–STING/JAK–STAT (e.g., MGF505–6R), context-dependent IFN responses can still induce ISGs such as CMPK2 at 2 dpi [49]. Moreover, ASFV’s mitochondrial targeting and redox/stress-granule remodeling mechanistically link CMPK2’s mitochondrial functions to ASFV inflammatory pathogenesis [40,50].

ZBP1 showed high centrality in the PPI network and was classified as a hub gene based on its Maximal Clique Centrality (MCC) score. ZBP1 is a type-I-IFN-inducible ISG that senses Z-form nucleic acids and can trigger RIPK3–MLKL–dependent necroptosis [43,51]. In the context of ASFV, recent work demonstrates that infection facilitates Z-DNA accumulation and assembly of the ZBP1–RIPK3–MLKL necrosome, activating macrophage necroptosis and restricting ASFV replication [52]. Consistent with these data, the up-regulation of ZBP1 at 2 dpi in our spleen dataset plausibly reflects sensing of ASFV-derived Z-nucleic acids and engagement of necroptotic signaling in infected macrophages to limit viral dissemination [53].

African swine fever is characterized in vivo by severe hemorrhagic splenomegaly, vascular/endothelial injury, and DIC-like coagulopathy, which are largely driven by massive activation and subsequent dysregulation of ASFV-infected macrophages/monocytes. These cells release high levels of TNF-α, IL-1β, IL-6 and other pro-inflammatory mediators that secondarily damage endothelium and promote vascular leakage and thrombosis. This sequence has been described in experimental and field infections, where macrophage-centered cytokine storms preceded or paralleled the appearance of hemorrhages and coagulopathy [35,54,55].

In our dataset, the 2 dpi pink module corresponds exactly to this early macrophage/innate antiviral burst (ISGs, RIG-I/MDA5, cGAS–STING, necroptosis genes such as ZBP1–RIPK3–MLKL). Such signatures fit well with prior reports showing that ASFV primarily targets splenic macrophages/monocytes and that infected or by-stander macrophages are the main source of inflammatory cytokines that underlie vascular lesions and lymphoid apoptosis [22,56]. In conclusion, the cyan and pink co-expression modules characterize the early host response to ASFV infection at 2 dpi. In particular, the pink module reflects a macrophage-driven antiviral and inflammatory program, marked by activation of ISGs, pattern-recognition signaling, and necroptotic pathways. The identification of CMPK2 and ZBP1 as central hub genes highlights their potential roles in coordinating mitochondrial stress responses and regulated cell death mechanisms during the early containment phase of infection. Taken together, these findings suggest that 2 dpi represents a critical window in which innate immune activation precedes the onset of vascular injury and coagulopathic pathology in acute ASFV, providing insights into early host–virus interactions warranting further investigation.

### 4.2. Modulation of TNF/TGF-β-Associated Gene Expression at 2 dpi

At 2 dpi, the red module exhibited coordinated downregulation of transcripts annotated to TNF- and TGF-β–associated signaling programs. Rather than indicating a lack of inflammatory activity, this pattern is consistent with the early regulatory phase of ASFV infection, during which infected macrophages initiate antiviral signaling while simultaneously altering inflammatory tone to delay overt tissue damage. Experimental ASFV studies have shown that the peak hemorrhagic and coagulopathic pathology generally emerges after 3–5 dpi, following the massive activation and dysregulation of splenic macrophages [57]. Thus, our 2 dpi sampling likely captures the stage prior to widespread vascular injury, in which cytokine responses are present but not yet translated into full histopathological damage. The enrichment of TGF-β/SMAD and neurodevelopment-associated regulatory clusters further supports this interpretation. TGF-β signaling is known to modulate innate immune responses, macrophage polarization, and tissue repair programs. The downregulation observed here may reflect host attempts to balance antiviral defense with protection of stromal and endothelial integrity at an early disease stage. Rather than reflecting suppression of immunity, these coordinated transcriptional shifts suggest a transitional immune–developmental regulatory state that precedes the inflammatory and hemorrhagic pathology characteristic of acute ASFV.

Taken together, the downregulation of the red module at 2 dpi likely represents a pre-hemorrhagic regulatory phase, in which spleen tissue maintains controlled immune signaling prior to the escalation of vascular and coagulopathic injury observed later in infection.

### 4.3. Upregulation of Signaling and Translation-Associated Modules at 5 dpi and Their Pathological Implications

By 5 dpi, which overlaps with the 4–6 dpi window when highly virulent ASFV strains begin to show marked splenic congestion, lymphoid depletion, and vascular/endothelial lesions in vivo, the co-expression patterns suggest a shift in cellular state toward signaling networks and protein-synthesis pathways that are typically observed during the later stage of ASFV infection. Rather than indicating complete immune shutdown, these transcriptional changes likely reflect host–cell reprogramming as viral replication increases.

The turquoise module showed significant enrichment in various intracellular signaling pathways. These functions are essential roles for cell migration, adhesion, cytoskeletal remodeling, tissue repair, and damage response, and they reflect host tissue remodeling in response to viral infection. The MAPK signaling pathway showed significant enrichment, indicating a strong association with viral replication. Previous studies have shown that ASFV activates the MAPK pathway to promote viral replication, and inhibition of this pathway markedly reduces viral proliferation [58]. Furthermore, cAMP is known as a key regulator of innate and adaptive immune cell function [59]. Previous studies have shown that cAMP signaling suppresses host immune responses and that microbes, including viruses, can exploit this pathway by enhancing cAMP activity to establish an immunosuppressive environment and facilitate immune evasion [60]. Among the genes shared by the MAPK pathway and the cAMP signaling pathway, PIFQ and AATK showed increased expression at 5 dpi (Figure 7). Therefore, the increased expression of the turquoise module at 5 dpi may reflect ASFV’s strategy to suppress host immune defenses and create a favorable intracellular environment for viral replication.

The brown module was primarily associated with ribosomal function and exhibited a strong positive correlation with infection at 5 dpi, a late stage of viral infection. Notably, these genes included in this pathway showed increased expression at 5 dpi (Figure 7). ASFV is known to hijack the host’s protein synthesis machinery to support its replication. During the early stage of infection, it activates factors that initiate viral protein production. Later, it brings ribosomes and translation components to viral factories, increasing protein production and virus replication [61]. The upregulation of ribosome-related genes reflects ASFV’s mobilization of the host translation system to optimize viral protein synthesis in the late phase of infection.

Notably, genes in the brown module identified by ClueGO/CluePedia were upregulated at 5 dpi (Figure 7), suggesting a late-stage reorganization of receptor composition and excitatory signaling in immune cells, which in turn regulates membrane potential and vesicle pH for secretion and endocytosis. This coordinated remodeling of ion channels, vesicle transport, and metabolism likely creates a cellular state that favors ASFV replication. Consistently, ASFV is known to enhance replication by modulating host energy and amino acid metabolism, relieving translational inhibition through viral proteins, and altering cholesterol transport to generate a permissive membrane environment for virion production [62,63,64,65]. Overall, these brown-module changes indicate that ASFV drives late-stage reprogramming of host membrane and metabolic pathways, highlighting potential targets to limit viral output.

Therefore, the turquoise and brown modules are activated during the late stage of ASFV infection (5 dpi), indicating a shift toward a suppressed immune response in the host. At the same time, signaling pathways and protein translation are reorganized under viral control, showing that the host cell environment is altered to promote viral replication.

Hemorrhagic lesions in ASFV have been associated with endothelial activation/damage, VEGF-linked vascular remodeling, and consumption coagulopathy [66,67]. At 5 dpi, the turquoise and brown modules showed enrichment of pathways related to cell adhesion, focal adhesion, Ca^2+^ and MAPK signaling, and vesicle/ion transport. Because this dataset represents bulk spleen tissue, these results should be interpreted as transcriptional correlations rather than direct evidence of endothelial pathology. However, the patterns we observe are consistent with microenvironmental remodeling processes reported during acute ASFV infection, and therefore likely reflect late-phase host cellular reprogramming that accompanies increasing viral replication.

### 4.4. Suppression of Antiviral and Metabolic Modules at 5 dpi

At 5 dpi, the blue module showed strong negative correlation with infection, indicating coordinated suppression of cellular protein synthesis and mitochondrial energy production (Figure 7).This transcriptomic pattern is consistent with previous reports that ASFV redirects host translation toward viral protein synthesis while limiting host-driven protein production [68,69].

Within this module, two hub genes, USP7 and EPRS1, displayed early upregulation at 2 dpi followed by marked suppression at 5 dpi (Figure 7). While direct targeting of USP7 by ASFV has not been conclusively shown, many DNA viruses hijack or modulate USP7 to favor infection—for example, HSV-1 ICP0 and EBV EBNA1 bind USP7 to remodel host antiviral and p53 pathways [70,71,72]. In ASFV, ubiquitin and SUMO circuits are directly rewired by viral factors—e.g., the ASFV E2 enzyme UBCv1/pI215L and the cysteine protease pS273R—to antagonize type-I IFN/JAK-STAT signaling and host translation [73,74,75]. Taken together, the down-regulation of USP7 at 5 dpi is consistent with ASFV-driven remodeling of ubiquitin/SUMO and translational programs, and with the observed broad suppression of blue-module processes (gene regulation, protein synthesis, energy metabolism), which would blunt immune function and tissue repair and ultimately favor viral persistence.

EPRS1, a component of the GAIT complex that translationally silences inflammatory mRNAs, [76,77] was also reduced at 5 dpi. Loss of GAIT-mediated repression has been linked to excessive cytokine output and macrophage hyperactivation, which aligns with the dysregulated inflammatory states observed in ASFV infection [76]. Thus, decreased EPRS1 expression may contribute to uncontrolled cytokine signaling and tissue injury during late infection. Importantly, ASFV infections exhibit robust cytokine induction/“storm-like” responses in macrophages and pigs [52,78], and ASFV actively rewires host translation/stress-response pathways—for example, pS273R cleaves G3BP1 to block stress-granule formation and facilitate replication [74]. Together, these observations provide a mechanistic rationale that reduced EPRS1 at late infection could dampen GAIT-mediated anti-inflammatory control, contributing to excess cytokine output and tissue damage in ASFV pathogenesis.

The pink module was activated at 2 dpi but became suppressed by 5 dpi, and Key hub genes within this module, such as CMPK2 and ZBP1, were also downregulated at 5 dpi. Previous studies have demonstrated that suppression of CMPK2 disrupts mitochondrial homeostasis in macrophages, leading to membrane depolarization, increased reactive oxygen species (ROS) production, and elevated expression of pro-inflammatory cytokines such as IL-1β, TNF-α, and IL-8 [79]. ZBP1 is an interferon-stimulated gene (ISG) that induces programmed cell death in virus-infected cells. The suppression of these immune-related genes at 5 dpi is likely caused by the action of ASFV-encoded proteins, such as EP152R and MGF360/505, which interfere with host defense mechanisms (Figure 7) [80,81]. This downregulation in the late stage of infection indicates impaired host immunity and facilitates viral replication. In conclusion, the suppression of the blue and pink modules at 5 dpi suggests that ASFV employs a multifaceted immune evasion strategy by simultaneously inhibiting host energy production, protein synthesis, and antiviral immune responses. Central genes such as EPRS1, USP7, CMPK2, and ZBP1 are likely key regulators of ASFV pathogenesis. Future functional validation of these targets may provide valuable insights into the mechanisms of ASFV infection and facilitate the development of effective therapeutic strategies.

In this study, we further confirmed that 2 days post-infection (dpi) represents a mechanistically distinct early stage of African swine fever virus (ASFV) infection. A previous study (Oh et al., 2024) reported that the NF-κB signaling pathway becomes activated at 3 dpi; however, by including the 2 dpi time point, we found that NF-κB–mediated innate immune responses were already robustly induced at this earlier stage [27]. Notably, at 2 dpi, not only NF-κB but also multiple innate immune pathways—including cGAS–STING, RIG-I/MDA5, interferon-stimulated genes (ISGs), and necroptosis-related programs—were strongly activated, and these pathways (except for NF-κB, ISGs) were not identified in the previous study. This discrepancy is likely due to the fact that our analysis focused specifically on the spleen, whereas the previous study analyzed nine different tissues, potentially diluting spleen-specific immune signatures.

Furthermore, by extending our analysis to 5 dpi, we identified a critical transition from early antiviral defense to late-stage immune collapse that was not fully captured in earlier datasets. At 5 dpi, NF-κB signaling and several other innate immune pathways were broadly suppressed, consistent with the inhibition of host protein synthesis, mitochondrial dysfunction, and metabolic collapse characteristic of terminal ASFV disease. These findings highlight 5 dpi as a unique and previously uncharacterized stage that captures the progression from robust early innate immune activation to profound late-stage immunosuppression. To directly link molecular findings with disease progression, we summarized clinical and transcriptomic changes across infection stages in a Supplementary Table (Supplementary Table S1). This table integrates body temperature, spleen viral load, key differentially expressed genes, and major enriched pathways at 0, 2, and 5 dpi. Early fever and moderate viral replication at 2 dpi corresponded with strong activation of antiviral pathways (cGAS–STING, RIG-I/MDA5, interferon-stimulated genes), whereas the severe clinical deterioration and high viral burden observed at 5 dpi aligned with transcriptomic signatures of suppressed protein synthesis, impaired mitochondrial function, and immunometabolic collapse. This integrated overview demonstrates that the transcriptional transitions identified in our study closely reflect the in vivo progression of acute ASFV infection. Taken together, these findings illustrate a clear transition from early antiviral activation to late stage immunometabolic collapse during ASFV infection. However, several limitations of this study should be acknowledged to properly contextualize these results, as detailed in the following subsection.

### 4.5. Study Limitations

Although this study primarily focused on transcriptomic analysis, supplementary clinical observations from a previous experiment [27] (Oh et al., 2024) showed increased body temperature and elevated spleen viral titers following infection, accompanied by clinical signs such as reduced alertness, diarrhea, and hemorrhagic manifestations (Supplementary Figures S1, S2 and Table 1). These clinical findings were broadly consistent with the transcriptomic signatures observed in this study, including the marked suppression of host protein synthesis and metabolic pathways at 5 dpi, which reflects the systemic pathological deterioration characteristic of the late stage of acute ASFV infection. However, the present study is limited by the absence of comprehensive clinical–pathological data across all time points, which prevents direct correlation analyses between disease progression and molecular responses. In addition, only two spleen samples were available at 5 dpi, reducing the statistical robustness of gene-expression estimates for this late time point. Therefore, interpretations of the 5 dpi results should be made with caution, and future studies with larger sample sizes and complete pathological datasets will be needed to validate these findings.

An additional consideration is that the transcriptomic interpretations presented here would benefit from further functional validation in future studies. Because this study relies solely on gene-expression signatures, additional experimental confirmation is required to establish causality. Future studies must introduce targeted analytical methods such as RT-qPCR, protein level measurements, and in vitro manipulation of key pathways to validate the mechanistic roles performed by the identified genes and modules in African swine fever virus (ASFV) pathogenesis.

## 5. Conclusions

We provide a time-resolved network view of ASFV infection in porcine spleen. Six co-expression modules showed clear temporal patterns across infection, reflecting an early (2 dpi) activation of innate antiviral responses followed by a late (5 dpi) suppression of immune and core cellular functions. Within these dynamics, CMPK2 and ZBP1 emerged as key genes associated with early mitochondrial innate sensing and necroptosis-linked antiviral defense, while EPRS1 and USP7 characterized later-stage downregulation of translation, ubiquitin-associated regulation, and cellular energy programs.

These findings suggest that ASFV infection proceeds through phase-specific transcriptional architectures, transitioning from early immune activation to later suppression of host protein synthesis, metabolism, and inflammatory control. However, our interpretations are limited by sample size, uneven sampling intervals, bulk-tissue profiling, and the use of a single viral strain. Further protein-level, cell-type–resolved, and functional validation will be necessary to confirm mechanistic roles and evaluate translational relevance. In summary, CMPK2 and ZBP1 represent promising candidates for early antiviral regulation, whereas EPRS1 and USP7 may serve as potential regulators of late immunometabolic and translational reprogramming during ASFV infection. These findings delineate dynamic spleen transcriptional responses to ASFV infection and suggest coordinated shifts in mitochondrial–innate and translational/ubiquitin pathways across infection stages. Further experimental and protein-level validation in independent and field-infected populations will be required to confirm mechanistic roles and assess potential translational relevance.

## Figures and Tables

**Table 1:** Summary Statistics of Quality control of sequenced reads: Results for 13 samples.

Metric	Mean	Standard Deviation	Minimum	Maximum
Input Reads Pairs	36,484,500	3,403,084	32,438,387	42,868,927
Both Surviving	35,227,179	3,469,893	31,174,125	41,644,734
Forward Only Surviving	1,089,212	266,983	517,700	1,719,073
Reverse Only Surviving	134,419	35,440	105,449	231,891
Dropped	33,691	9973	23,479	63,752

**Table 2:** WGCNA modules with enriched KEGG and GO (BP) terms and key representative genes.

Module	Enriched KEGG Pathway and Gene Ontology (Biological Process) Terms	Adjusted *p*-Value	Genes
Pink	Influenza A Hepatitis C necroptosis RIG-I-like receptor signaling Toll-like receptor signaling cyt solic DNA sensing pathway defense response to virus (GO:0051607) innate immune response (GO:0045087) response to virus (GO:0009615)	8.11 × 10^−7^ 1.39 × 10^−5^ 1.30 × 10^−3^ 1.67 × 10^−4^ 1.30 × 10^−3^ 1.90 × 10^−4^ 1.73 × 10^−18^ 1.69 × 10^−13^ 1.66 × 10^−17^	*MLKL*, *IRF3*, *RSAD2*, *OAS2*, *OASL*, *PDE12*, *MORC3*, *MX2*, *MX1*, *IRF7*, *IFIH1*, *TRIM65*, *DHX58*, *ISG20*, *RIGI* (*DDX58*), *CXCL10*, *BST2*, *PLSCR1*, *ISG15*, *IFIT3*, *ZBP1*, *IFI44L*, *PARP12*, *CMPK2*
Cyan	Huntington’s disease selenocompound metabolism amyotrophic lateral sclerosis (ALS)	0.05 0.05 0.05	
Red	TNF signaling	0.026	*DNAJC27*, *UBA3*, *GJA1*, *TPM1*, *GDF5*, *HOXA11*
Turquoise	Huntington’s disease selenocompound metabolism amyotrophic lateral sclerosis (ALS)	0.05 0.05 0.05	*MNX1*, *DMRT3*, *OTX1*, *SP8*, *CAMK2B*, *SOX14*, *TRPM8*, *CHRNA10*, *DRGX*
Brown	ribosome	1.99035 × 10^−2^	*NTRK3*, *DCH24*, *CDH24*, *CDH13*, *CAMSAP1*, *CHRNA5*, *FBLN2*, *CDH18*, *LMO1*, *CHRNA4*, *GABRG2*, *GABRD*, *KCNJ9*, *SHISA7*, *SLC26A3*, *CLIC6*
blue	oxidative phosphorylation ribosome function thermogenesis Alzheimer’s disease Parkinson’s disease Huntington’s disease protein translation (GO:0006412) ribosome biogenesis (GO:0042254) rRNA metabolic process (GO:0016072) ribonucleoprotein complex bi genesis (GO:0022613)	5.9663 × 10^−14^ 9.4239 × 10^−28^ 2.4564 × 10^−11^ 6.293 × 10^−12^ 3.2788 × 10^−21^ 8.0229 × 10^−16^ 2.2162 × 10^−11^ 7.7883 × 10^−10^ 4.7359 × 10^−8^ 2.2162 × 10^−11^	*RPS11*, *RPS5*, *RPS12*, *RPL14*, *RPS4X*, *RPS23*, *RPL26L1*, *EIF4A3*, *RPS16*, *RPS21*, *RPS27L*, *RSL24D1*, *RPS7*, *RPL35*, *RPLP0*, *RPSA*, *RPS24*, *RPL22*, *RPS19BP1*, *TMEM182*, *B3GNT2*, *EIF2AK2*, *RCN2*, *EIF2AK3*, *KDELR2*, *EIF2A*, *HSD17B10*, *RPLP1*, *PPP2R1B*, *EIF2S2*, *ARHGEF18*, *EPRS1*, *USP7*, *WDR12*, *HNRNPM*, *DDX2*, *PRPF*, *EIF4A1*

## Data Availability

The transcriptomic datasets analyzed in this study are publicly available in the NCBI GEO database under accession number GSE230340. No new datasets were generated.

## Supplementary Materials

The following supporting information can be downloaded at: https://www.mdpi.com/article/10.3390/life15121844/s1, Figure S1: Intrasplenic viral titers in ASFV-infected pigs at 2 and 5 days post-infection (dpi); Figure S2: Changes in body temperature in African swine fever virus (ASFV)-infected pigs; Table S1: Clinical progression patterns, viral load, body temperature, major modules, key genes, and major enrichment pathways at each stage of ASFV infection.
