# Supplementary material for: Dynamic Gene Network Alterations and Identification of Key Genes in the Spleen During African Swine Fever Virus (ASFV) Infection
Source: Life (Basel). 2025 Nov 30;15(12):1844. doi: 10.3390/life15121844 (PMC12734043; doi:10.3390/life15121844)
Supplement: Supplementary file 1 [file life-15-01844-s001.zip › Supplementary_Table S1.pdf]

Supplementary Table S1

Clinical progression, viral load dynamics, and spleen transcriptomic modules across ASFV infection stages (0, 2, and 5 dpi).

Time point	Clinical observations	Viral load (Mean value)	Body Temperature (Mean value)	Key modules and hub genes	Major enriched pathways
0	Normal body temperature; no clinical abnormalities		38.76 ℃		
2	Mild fever; no apparent clinical abnormalities	240,897	39.55 ℃	Activation of pink and cyan modules; hub genes CMPK2 and ZBP1 (ISGs: MX1, ISG15, IFIT3, IRF7)	Innate antiviral and inflammatory pathways: RIG-I-like receptor (ssc04622), Toll-like receptor signaling (ssc04620), cytosolic DNA-sensing (ssc04623), necroptosis (ssc04217); defense response to virus (GO:0051607), innate immune response (GO:0045087); oxidative-stress/mitochondrial pathways (ssc00190)
5	Spleen swelling, infarction; mesenteric lymph nodes hemorrhagic; inguinal swollen hemorrhage; mandibular swollen hemorrhagic; kidney hemorrhage pins	5,899,354	40.5 ℃	Activation of turquoise and brown modules with late-stage reprogramming; suppression of blue and pink modules. Key hub genes: EPRS1, USP7, CMPK2, ZBP1	Downregulation of innate immunity; remodeling of protein translation (ssc03010)
