# Supplementary figures and images for: Dynamic Gene Network Alterations and Identification of Key Genes in the Spleen During African Swine Fever Virus (ASFV) Infection
Source: Life (Basel). 2025 Nov 30;15(12):1844. doi: 10.3390/life15121844 (PMC12734043; doi:10.3390/life15121844)

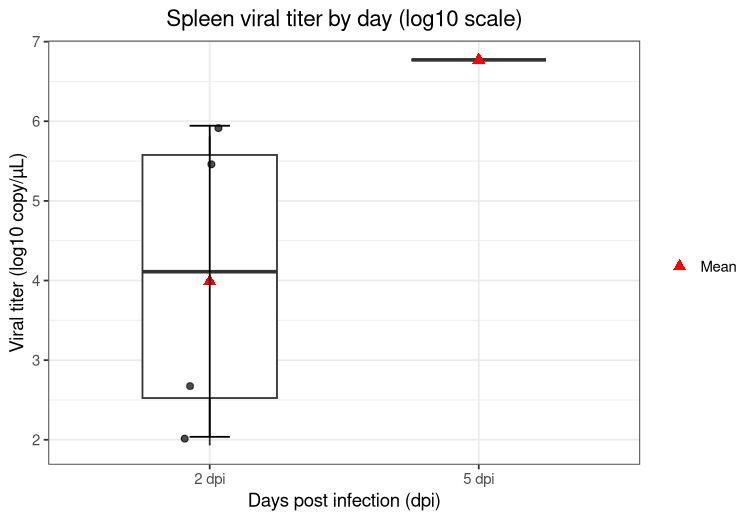

Supplement: Supplementary file 1 [file life-15-01844-s001.zip › Supplementary_Figure S1.png]

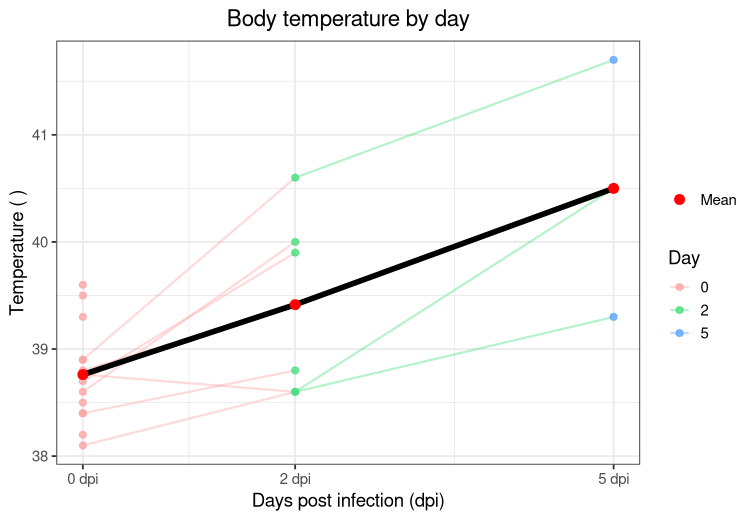

Supplement: Supplementary file 1 [file life-15-01844-s001.zip › Supplementary_Figure S2.png]
